# Association of social distancing and face mask use with risk of COVID-19

**DOI:** 10.1038/s41467-021-24115-7

**Published:** 2021-06-18

**Authors:** Sohee Kwon, Amit D. Joshi, Chun-Han Lo, David A. Drew, Long H. Nguyen, Chuan-Guo Guo, Wenjie Ma, Raaj S. Mehta, Fatma Mohamed Shebl, Erica T. Warner, Christina M. Astley, Jordi Merino, Benjamin Murray, Jonathan Wolf, Sebastien Ourselin, Claire J. Steves, Tim D. Spector, Jaime E. Hart, Mingyang Song, Trang VoPham, Andrew T. Chan

**Affiliations:** 1 Clinical and Translational Epidemiology Unit, Massachusetts General Hospital and Harvard Medical School, Boston, MA USA; 2 Division of Gastroenterology, Massachusetts General Hospital and Harvard Medical School, Boston, MA USA; 3 Department of Biostatistics, Harvard T.H. Chan School of Public Health, Boston, MA USA; 4 Department of Medicine, Li Ka Shing Faculty of Medicine, University of Hong Kong, Hong Kong, China; 5 Medical Practice Evaluation Center, Massachusetts General Hospital and Harvard Medical School, Boston, MA USA; 6 Harvard/MGH Center on Genomics, Vulnerable Populations, and Health Disparities, Massachusetts General Hospital, Boston, MA USA; 7 Division of Endocrinology and Computational Epidemiology, Boston Children’s Hospital and Harvard Medical School, Boston, MA USA; 8 Broad Institute of MIT and Harvard, Cambridge, MA USA; 9 Diabetes Unit, Center for Genomic Medicine, Massachusetts General Hospital, Boston, MA USA; 10 Programs in Metabolism and Medical & Population Genetics, Broad Institute of MIT and Harvard, Cambridge, MA USA; 11 Department of Medicine, Harvard Medical School, Boston, MA USA; 12 School of Biomedical Engineering & Imaging Sciences, King’s College London, London, UK; 13 Zoe Limited, London, UK; 14 Department of Twin Research and Genetic Epidemiology, King’s College London, London, UK; 15 Channing Division of Network Medicine, Department of Medicine, Brigham and Hospital and Harvard Medical School, Boston, MA USA; 16 Exposure, Epidemiology and Risk Program, Department of Environmental Health, Harvard T.H. Chan School of Public Health, Boston, MA USA; 17 Department of Epidemiology, Harvard T.H. Chan School of Public Health, Boston, MA USA; 18 Department of Nutrition, Harvard T.H. Chan School of Public Health, Boston, MA USA; 19 Epidemiology Program, Division of Public Health Sciences, Fred Hutchinson Cancer Research Center, Seattle, WA USA; 20 Department of Epidemiology, University of Washington School of Public Health, Seattle, WA USA; 21 Department of Immunology and Infectious Disease, Harvard T.H. Chan School of Public Health, Boston, MA USA

**Keywords:** SARS-CoV-2, Viral infection, Lifestyle modification, Epidemiology

## Abstract

Given the continued burden of COVID-19 worldwide, there is a high unmet need for data on the effect of social distancing and face mask use to mitigate the risk of COVID-19. We examined the association of community-level social distancing measures and individual face mask use with risk of predicted COVID-19 in a large prospective U.S. cohort study of 198,077 participants. Individuals living in communities with the greatest social distancing had a 31% lower risk of predicted COVID-19 compared with those living in communities with poor social distancing. Self-reported ‘always’ use of face mask was associated with a 62% reduced risk of predicted COVID-19 even among individuals living in a community with poor social distancing. These findings provide support for the efficacy of mask-wearing even in settings of poor social distancing in reducing COVID-19 transmission. Despite mass vaccination campaigns in many parts of the world, continued efforts at social distancing and face mask use remain critically important in reducing the spread of COVID-19.

## Abstract (web summary)

Estimating the effectiveness of COVID-19 control measures requires large prospective data including symptoms and personal risk factors. Here, the authors used data from smartphone-based application and found that individual face mask use was associated with a 64% reduced risk of COVID-19 symptoms.

## Introduction

The COVID-19 pandemic is ongoing and new COVID-19 cases continue to rise globally^1^. As of March 14, 2021, over 119 million global cases of COVID-19 and nearly 2.6 million global deaths have been documented^1,2^ Although mass vaccination programs started in December 2020 in high-income countries^3^, only 439 million vaccine doses, equivalent to 5.7 doses for every 100 people, have been administered worldwide so far^4^. Moreover, inequities in vaccine allocation and delivery among lower-income countries remain a significant threat to worldwide control of the pandemic^5^. Current estimates suggest that it will be at least 2023 until there are sufficient vaccine doses to cover the world’s population^6^. Therefore, nonpharmaceutical interventions, including social distancing and face mask use, will continue to play a key role to mitigate the risk of COVID-19 for the foreseeable future^7,8^. Furthermore, social distancing and face mask use remain strongly recommended even after vaccination^9^ because vaccines cannot completely prevent infection^10^ and their role in preventing asymptomatic transmission of COVID-19 is uncertain. Therefore, given the continued burden of COVID-19, there is a high unmet need for real-world data to investigate the effect of social distancing and face mask use to mitigate the risk of COVID-19.

To date, much of the evidence on the efficacy of social distancing and face mask use is based on modeling using mostly community-level data in relation to disease burden as assessed through testing, hospitalizations, or mortality^11–23^. Such studies are unable to concurrently account for personal risk factors for infection or optimally assess the latency between social distancing or face mask-use interventions and infection rates given the significant lag between the onset of symptoms, testing, and medical care. Moreover, most evidence with individual-level data includes a relatively limited number of participants^24–28^. Here, we conducted a large size of a prospective study in the US using a smartphone-based application that collected self-reported, individual-level information on COVID-19-like symptoms, face mask use, and other personal risk factors, in combination with community-level social-distancing measures to investigate the relative effectiveness of social distancing and face mask-use policies with the risk of COVID-19.

## Results

Between March 29 and July 16, 2020, we enrolled 277,798 participants who provided baseline information. We excluded 79,721 individuals who did not live in a county with available Unacast data, reported any symptoms or a positive COVID-19 test at enrollment, had <24 h of follow-up time, or who reported a positive COVID-19 test or symptoms of predicted COVID-19 within 24 h of enrollment. This left 198,077 participants in our prospective inception cohort, in which we subsequently documented 4488 cases of predicted COVID-19 over 11,428,442 person-days of follow-up for the social-distancing analysis. Among 198,077 participants, we excluded 63,480 who did not answer to face mask-use questions for the face mask-use analysis. This left 134,597 participants in our prospective inception cohort, in which we subsequently documented 1194 cases of predicted COVID-19 over 4,209,237 person-days of follow-up for the face mask-use analysis. Compared to others, individuals who lived in communities with poor social distancing (Grade = F) at baseline were younger, more likely to be male, more likely to smoke currently, have less lung disease, had more interaction with suspected or documented COVID-19 individuals, and more likely to live in areas with higher neighborhood deprivation index (Table 1). In contrast, individuals living in communities with excellent social distancing (Grade = A/B) were older and more likely to live in areas with lower population density (Table 1).

**Table 1:** Baseline characteristics of study participants according to overall social distancing grade.

Overall social distance grade^a^		Overall	Poor (F)	Fair (D)	Good (C)	Excellent (A/B)
		*n* = 198,077	*n* = 28,439	*n* = 63,331	*n* = 92,640	*n* = 13,667
Age (years), %						
	<25	7.8	10.2	8.1	7.0	6.3
	25–34	9.5	10.0	8.6	10.2	7.6
	35–44	13.6	14.6	13.5	13.7	11.2
	45–54	14.9	15.6	15.1	14.7	13.5
	55–64	20.3	19.2	21.2	20.0	19.6
	≥65	34	30.3	33.4	34.4	41.8
	Missing	0.0	0.0	0.0	0.0	0.0
Male sex, %		35.2	39.5	35.3	33.8	34.7
Race/ethnicity^b^, %						
	White, non-Hispanic	83.9	84.2	84.2	83.3	84.6
	Hispanic/Latinx	5.6	6.1	5.5	5.6	4.7
	Black	2.9	3.0	3.1	2.9	1.2
	Asian	3.6	2.7	3.3	4.5	4.3
	Mixed/other race	2.9	2.8	2.9	2.7	3.9
	Prefer not to say	0.8	0.8	0.7	0.8	0.9
	Missing	0.3	0.4	0.3	0.2	0.4
Current smoker, %		5	6.2	5.4	4.5	4.0
	Missing	0.1	0.0	0.1	0.1	0.0
Comorbidities, %						
	Diabetes	5.8	5.0	6.5	5.7	4.9
	Heart disease	6.2	6.5	6.3	6.0	6.3
	Lung disease	11.5	7.7	12.0	12.2	12.1
	Kidney disease	1.5	1.6	1.5	1.5	1.4
Population density, %						
	Quartile 1	25.5	23.9	28.2	20.4	50.7
	Quartile 2	24.7	30.1	27.4	21.6	22.5
	Quartile 3	24.5	27.6	25.4	24.8	11.8
	Quartile 4	24.7	17.6	18.3	32.8	14.2
	Missing	0.6	0.7	0.7	0.4	0.8
Frontline healthcare worker, %		9.3	7.6	9.5	9.9	8.7
Interaction with suspected or documented Covid-19, %		8.9	10.4	8.4	8.9	8.1
Health problems requiring stay-at-home^c^, %		4.6	5.5	4.9	4.2	3.8
Regular use mobility aid^d^, %		2	2.4	2.0	1.9	1.9
Health problems limiting activities^e^, %		8.5	9.6	8.9	8.1	7.9
Neighborhood Deprivation Index, %						
	Quartile 1	25.5	18.7	18.4	32.1	27.6
	Quartile 2	23.1	23.5	23.0	23.4	20.8
	Quartile 3	24.3	25.6	25.8	22.4	26.7
	Quartile 4	26.0	30.9	31.6	21.2	23.5
	Missing	1.1	1.4	1.2	0.9	1.4

^a^Overall social distancing grades are denoted as Poor (F grade), Fair (D grade), Good (C grade), and Excellent (A + B grade) from Unacast mobility data.

^b^The proportion of race was calculated among the participants who received the race question which was added at April 18, 2020.

^c^Asked as “In general, do you have any health problems that require you to stay at home”?

^d^Asked as “Do you regularly use a stick, walking frame or wheelchair to get about”?

^e^Asked as “In general, do you have any health problems that require you to limit your activities”?

### Risk of predicted COVID-19 according to overall community social distancing grade at various time lags

**Fig. 1:**
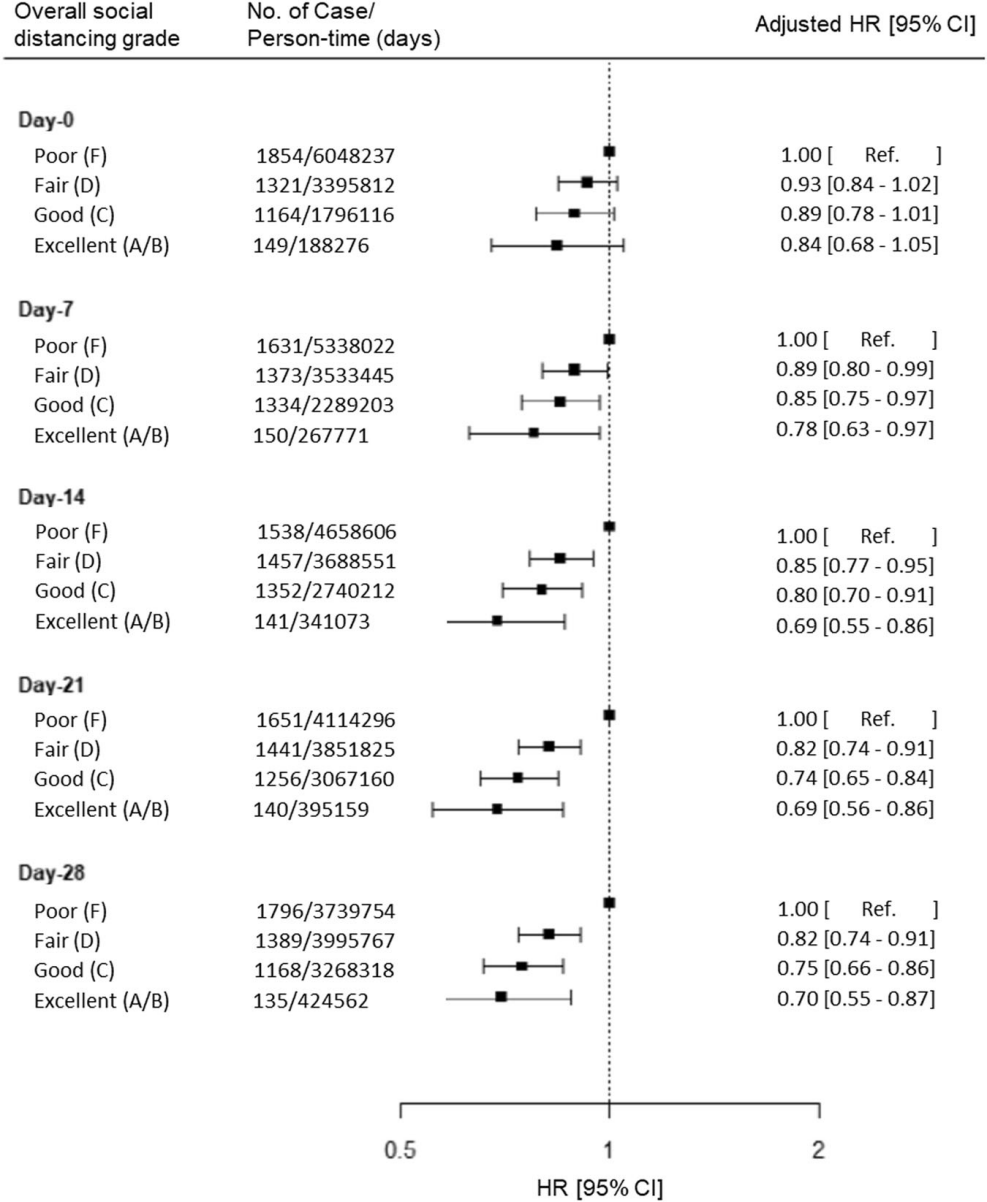
Risk of predicted Covid-19 according to living in a community with overall social-distancing grade at various time lags. Overall social-distancing grades are denoted as Poor (F grade), Fair (D grade), Good (C grade), and Excellent (A + B grade). Overall social-grade categories (A, B, C, D, and F) are provided by Unacast. Cox proportional hazards regression models were used to calculate adjusted HRs and 95% CIs of predicted COVID-19. Adjusted models were stratified by age (<25, 25–34, 35–44, 45–54, 55–64, ≥65), state, and calendar date at study entry and further adjusted for race (White, Black, Asian, or other), sex (male or female), population density of residence (quartiles), current smoking, frontline healthcare worker, interaction with suspected or documented Covid-19, history of diabetes, heart disease, lung disease, and kidney disease (each yes or no). HR hazard ratio, CI confidence interval.

To test the association between community-level social distancing and risk of subsequent predicted COVID-19, we evaluated lag times of 7–28 days. Living in a community with a greater social-distancing grade (F to A/B) was associated with a lower risk of predicted COVID-19 for all lag times evaluated (Table 2). The maximal association was first observed with a fourteen-day lag and the benefit plateaued beyond that time period (Fig. 1). Compared to participants living in communities with overall poor social distancing (Grade = F), the adjusted HRs for predicted COVID-19 at 14 days were 0.85 (95% CI 0.77–0.95) for fair (Grade = D), 0.80 (95% CI 0.70–0.91) for good (Grade = C), and 0.69 (95% CI 0.55–0.86) for excellent (Grade = A/B) social distancing (*P*_linear-trend_ < 0.001) after adjusting for personal risk factors for COVID-19 (Table 2). There was a negative but not statistically significant association with a 0-day lag. When we further adjusted for county-level test-positive COVID-19 incidence in the community at the time of assessment for the social-distancing measures, we observed similar results (adjusted HR, 0.67; 95% CI 0.53–0.85) for excellent social distancing (Grade = A/B) compared to participants living in communities with overall poor social distancing (Grade = F). For subsequent analyses, we focused on models using a fourteen-day latency since the reduction in predicted COVID-19 appeared maximal at 14 days, and this is considered a plausible interval for exposure to symptom-based disease prediction.

**Table 2:** Risk of predicted Covid-19 according to living in a community with overall social-distancing grade at various time lags.

Overall social distance grade^a^	Poor (F)	Fair (D)	Good (C)	Excellent (A/B)	*P* value for trend^b^
*Day—0*					
No. of cases/person-time (days)	1854/6,048,237	1321/3,395,812	1164/1,796,116	149/188,276	
Model 1 HR (95% CI)^c^	1 [Reference]	0.92 (0.83–1.02)	0.89 (0.78–1.01)	0.86 (0.69–1.06)	0.06
Model 2 HR (95% CI)^d^	1 [Reference]	0.93 (0.84–1.02)	0.89 (0.78–1.01)	0.84 (0.68–1.05)	0.06
*Day—7*					
No. of cases/person-time (days)	1631/5,338,022	1373/3,533,445	1334/2,289,203	150/267,771	
Model 1 HR (95% CI)	1 [Reference]	0.90 (0.81–1.00)	0.86 (0.76–0.98)	0.77 (0.62–0.96)	0.01
Model 2 HR (95% CI)	1 [Reference]	0.89 (0.80–0.99)	0.85 (0.75–0.97)	0.78 (0.63–0.97)	0.01
*Day—14*					
No. of cases/person-time (days)	1538/4,658,606	1457/3,688,551	1352/2,740,212	141/341,073	
Model 1 HR (95% CI)	1 [Reference]	0.85 (0.77–0.95)	0.79 (0.70–0.90)	0.68 (0.54–0.84)	1.03 × 10^−4^
Model 2 HR (95% CI)	1 [Reference]	0.85 (0.77–0.95)	0.80 (0.70–0.91)	0.69 (0.55–0.86)	2.61 × 10^−4^
*Day—21*					
No. of cases/person-time (days)	1651/4,114,296	1441/3,851,825	1256/3,067,160	140/395,159	
Model 1 HR (95% CI)	1 [Reference]	0.82 (0.74–0.91)	0.74 (0.65–0.84)	0.68 (0.55–0.85)	3.92 × 10^−6^
Model 2 HR (95% CI)	1 [Reference]	0.82 (0.74–0.91)	0.74 (0.65–0.84)	0.69 (0.56–0.86)	7.03 × 10^−6^
*Day—28*					
No. of cases/person-time (days)	1796/3,739,754	1389/3,995,767	1168/3,268,318	135/424,562	
Model 1 HR (95% CI)	1 [Reference]	0.82 (0.74–0.90)	0.75 (0.66–0.86)	0.69 (0.55–0.86)	1.46 × 10^−6^
Model 2 HR (95% CI)	1 [Reference]	0.82 (0.74–0.91)	0.75 (0.66–0.86)	0.70 (0.55–0.87)	1.94 × 10^−6^

*HR* hazard ratio, *CI* confidence interval.

Cox proportional hazards regression models were used to calculate HRs and 95% CIs.

^a^Overall social-distancing grades are denoted as Poor (F grade), Fair (D grade), Good (C grade), and Excellent (A + B grade). Overall social-grade categories (A, B, C, D, and F) are provided by Unacast.

^b^Two-sided *P* values for trend were calculated using the median value of each category as a continuous variable.

^c^Model 1 was stratified by age (<25, 25–34, 35–44, 45–54, 55–64, or ≥65), state, and calendar date at study entry.

^d^Model 2 was stratified by age (<25, 25–34, 35–44, 45–54, 55–64, ≥65), state, and calendar date at study entry and further adjusted for race (White, Black, Asian, or other), sex (male or female), population density of residence (quartiles), current smoking, frontline healthcare worker, interaction with suspected or documented Covid-19, history of diabetes, heart disease, lung disease, and kidney disease (each yes or no).

### Risk of predicted COVID-19 according to community social-distancing metrics and demographics

We also assessed the three individual components of the Unacast social-distancing grade: including average distance traveled, nonessential visitation, and human encounters (Table 3). Reduction in average distance traveled (adjusted HR, 0.78; 95% CI 0.65–0.92 < 25% versus >55%) and nonessential visitation (adjusted HR, 0.79; 95% CI 0.70–0.89 < 55% versus >65%) were both associated with lower risk of predicted COVID-19. The reduction in human encounters, based on phone-to-phone proximity measures, was not associated with lower risk of predicted Covid-19. In subgroup analyses, the association of social-distancing grade and COVID-19 appeared to differ according to age (*P*_interaction_ = 0.001). The association of Excellent (A/B) social distancing and the risk of predicted COVID-19 compared to Poor (F) was the greatest among the middle-age participants (35–55 years, adjusted HR, 0.47; 95% CI 0.26–0.84), than among younger (age < 35 years) or older participants (>55). We assessed for effect modification by other demographic including race, sex, and health problems limiting activities, and found no significant interactions between social-distancing grades and these factors (all *P*_interaction_ > 0.05; Supplementary Table 2). In addition, despite the limited power, we found a protective but not statistically significant association between community social distancing and risk of a positive COVID-19 test (Supplementary Table 4).

**Table 3:** Risk of predicted Covid-19 within 14 days according to individual metrics of social distancing^a^.

Social distance grade^b^	Poor (F)	Fair (D)	Good (C)	Excellent (A/B)	*P* value for trend^c^
*Metric 1: Percent reduction in average distance traveled*					
	<25%	25–40%	40–55%	>55%	
No. of cases/person-time (days)	1421/4,165,799	1233/3,293,375	1352/3,001,925	482/967,343	
Model 1 HR (95% CI)^d^	1 [Reference]	0.84 (0.76–0.93)	0.78 (0.69–0.88)	0.82 (0.70–0.96)	6.98 × 10^−4^
Model 2 HR (95% CI)^e^	1 [Reference]	0.84 (0.75–0.93)	0.77 (0.68-0.88)	0.78 (0.65–0.92)	4.33 × 10^−4^
*Metric 2: Percent reduction in nonessential visitation*					
	<55%	55–60%	60–65%	>65%	
No. of cases/person-time (days)	2164/6,350,546	445/1,151,909	533/1,174,359	1255/2,486,950	
Model 1 HR (95% CI)	1 [Reference]	0.84 (0.75–0.95)	0.85 (0.75–0.96)	0.79 (0.71–0.88)	1.58 × 10^−5^
Model 2 HR (95% CI)	1 [Reference]	0.84 (0.75–0.95)	0.85 (0.76–0.97)	0.79 (0.70–0.89)	4.84 × 10^−5^
*Metric 3: Percent reduction in human encounters*					
	<40%	74–40%	82–74%	>82%	
No. of cases/person-time (days)	3409/8,640,799	441/1,101,671	153/418,805	485/1,267,167	
Model 1 HR (95% CI)	1 [Reference]	1.01 (0.90–1.12)	0.99 (0.83–1.18)	0.96 (0.86–1.06)	0.59
Model 2 HR (95% CI)	1 [Reference]	1.02 (0.91–1.14)	1.00 (0.84–1.20)	0.95 (0.84–1.08)	0.77
*Overall social-distancing grade*^f^					
No. of cases/person-time (days)	1538/4,658,606	1457/3,688,551	1352/2,740,212	141/341,073	
Model 1 HR (95% CI)	1 [Reference]	0.85 (0.77–0.95)	0.79 (0.70–0.90)	0.68 (0.54–0.84)	1.03 × 10^−4^
Model 2 HR (95% CI)	1 [Reference]	0.85 (0.77–0.95)	0.80 (0.70–0.91)	0.69 (0.55–0.86)	2.61 × 10^−4^

*HR* hazard ratio, *CI* confidence interval.

Cox proportional hazards regression models were used to calculate HRs and 95% CIs.

^a^Day—14 is applied for models.

^b^Social-distancing grades are denoted as Poor (F grade), Fair (D grade), Good (C grade), and Excellent (A + B grade). The cutoffs for Metric 1, 2, and 3 and overall social-grade categories (A, B, C, D, and F) are provided by Unacast.

^c^Two-sided *P* values for trend were calculated using the median value of each category as a continuous variable.

^d^Model 1 was stratified by age (<25, 25–34, 35–44, 45–54, 55–64, or ≥65), state, and calendar date at study entry.

^e^Model 2 was stratified by age (<25, 25–34, 35–44, 45–54, 55–64, ≥65), state, and calendar date at study entry and further adjusted for the race (White, Black, Asian, or other), sex (male or female), population density of residence (quartiles), current smoking, frontline healthcare worker, interaction with suspected or documented Covid-19, history of diabetes, heart disease, lung disease, and kidney disease (each yes or no).

^f^The overall grade was calculated based on Metric 1, Metric 2, and Metric 3 as the average between the three numeric grades by Unacast.

Furthermore, to evaluate whether the impact of social distancing on the risk of predicted COVID-19 was modified by local transmissibility, we performed subgroup analysis according to Rt. During the epidemic slowing/maintenance period (Rt ≤ 1.0), compared to participants living in communities with overall poor social distancing (Grade = F), the adjusted HRs for predicted COVID-19 were 0.88 (95% CI 0.76–1.02) for fair (Grade = D), 0.79 (95% CI 0.66–0.95) for good (Grade = C), and 0.63 (95% CI 0.47–0.85) for excellent (Grade = A/B) social distancing (*P*_linear-trend_ = 0.002) after adjusting for personal risk factors for COVID-19 (Supplementary Table 6). This trend was also observed with similar magnitudes albeit with no statistical significance (*P*_linear-trend_ = 0.11) during the epidemic growth period (Rt > 1.0).

### Risk of predicted COVID-19 according to personal face mask use

We examined the association between self-reported personal face mask use and risk of predicted COVID-19 among the 134,597 participants who provided this information.

Compared to individuals who wore face masks none of the time, the adjusted HRs for predicted COVID-19 were 0.27 (95% CI 0.19–0.39) for individuals who wore face masks sometimes, 0.34 (95% CI 0.27–0.43) for individuals who wore face masks most of the time, and 0.36 (95% CI 0.30–0.44) for individuals who wore face masks always (*P*_linear-trend_ < 0.001) after adjusting for personal risk factors for COVID-19 (Table 4). Individuals who reported frequent face mask use were observed to have a reduced risk of predicted COVID-19 even in communities with poor social distancing. Among the individuals living in communities with poor social-distancing grade, the adjusted HRs for predicted COVID-19 were 0.27 (95% CI 0.18–0.41) for individuals who wore face masks sometimes, 0.38 (95% CI 0.30–0.48) for individuals who wore face masks most of the time, and 0.38 (95% CI 0.31–0.46) for individuals who wore face mask always (*P*_linear-trend_ < 0.001) compared to individuals who wore face masks none of the time (Table 4). The results remained similar after additional adjustment for actual COVID-19 incidence. Furthermore, observed associations were not substantially different when analyses were restricted to participants living in Texas, Arizona, California, and Florida, states which were among the states in which social-distancing policy was relaxed earlier during the initial phase of the pandemic.

**Table 4:** Personal use of a face mask outside the home and risk of predicted Covid-19.

		Frequency of personal use of a face mask^a^				
		None of the time	Sometimes	Most of the time	Always	*P* for trend^b^
*Overall*						
	No. of cases/person-time (days)	813/2,488,940	42/197,995	115/530,749	224/991,553	
	Model 1 HR (95% CI)^c^	1 [Reference]	0.28 (0.20–0.40)	0.33 (0.27–0.42)	0.35 (0.30–0.42)	1.29 × 10^−33^
	Model 2 HR (95% CI)^d^	1 [Reference]	0.27 (0.19–0.39)	0.34 (0.27–0.43)	0.36 (0.30–0.44)	7.59 × 10^−32^
*According to overall social distance grade of the community*^e^						
Non-poor (A/B/C/D)						
	No. of cases/person-time (days)	161/638,338	9/49,268	14/128,343	33/242,976	
	Model 1 HR (95% CI)	1 [Reference]	0.26 (0.11-0.62)	0.24 (0.13–0.45)	0.26 (0.16–0.43)	1.70 × 10^−9^
	Model 2 HR (95% CI)	1 [Reference]	0.27 (0.11-0.66)	0.25 (0.13–0.48)	0.28 (0.17-0.45)	6.99 × 10^−9^
Poor (F)						
	No. of cases/person-time (days)	652/1,850,602	33/148,727	101/402,406	191/748,577	
	Model 1 HR (95% CI)	1 [Reference]	0.28 (0.19–0.42)	0.36 (0.29–0.46)	0.37 (0.30–0.45)	5.70 × 10^−26^
	Model 2 HR (95% CI)	1 [Reference]	0.27 (0.18–0.41)	0.38 (0.30–0.48)	0.38 (0.31–0.46)	2.58 × 10^−24^

*HR* hazard ratio, *CI* confidence interval.

Cox proportional hazards regression models were used to calculate HRs and 95% CIs.

^a^Use of a face mask was collected from 139,690 participants beginning on June 12, 2020 based on the query “In the last week, did you wear a face mask when outside the house?”.

^b^Two-sided *P* values for trend were calculated as an ordinal variable.

^c^Model 1 was stratified by age (<25, 25–34, 35–44, 45–54, 55–64, or ≥65), state, and calendar date at study entry.

^d^Model 2 was stratified by age (<25, 25–34, 35–44, 45–54, 55–64, ≥65), state, and calendar date at study entry and further adjusted for race (White, Black, Asian, or other), sex (male or female), population density (quartiles), current smoking, frontline healthcare worker, interaction with suspected or documented Covid-19, history of diabetes, heart disease, lung disease, and kidney disease (each yes or no).

^e^Overall social-distancing grades are denoted as Poor (F grade), Fair (D grade), Good (C grade), and Excellent (A + B grade). Overall social-grade categories (A, B, C, D, and F) are provided by Unacast.

In subgroup analyses, we assessed for effect modification by demographic factors including race, sex, and health problems limiting activities (Supplementary Table 3). Despite no statistical evidence of heterogeneity, we observed that compared to individuals who wore face mask none of the time, individuals who always wore face mask appeared to have a lower risk of predicted COVID-19 if they were younger, had interacted with suspected or documented COVID-19 patients, regularly use a mobility aid, or had health problems that limited activities of daily living. In addition, despite the limited power, we found a similar association between face mask use and the risk of a positive COVID-19 test (Supplementary Table 5). Finally, the association of face mask use with predicted COVID-19 did not appear to substantially different according to Rt (Supplementary Table 7).

### Risk of predicted COVID-19 with social distancing and face mask use after adjusting for socioeconomic status

To account for socioeconomic status, we examined the association of social distancing and face mask use with the risk of COVID-19 after additionally adjusting for the neighborhood deprivation index. For social distance analysis, compared to participants living in communities with overall poor social distancing (Grade = F), the adjusted HRs for predicted COVID-19 were 0.87 (95% CI 0.78–0.97) for fair (Grade = D), 0.85 (95% CI 0.74–0.97) for good (Grade = C), and 0.75 (95% CI 0.60–0.93) for excellent (Grade = A/B) social distancing (*P*_linear-trend_ = 0.01) after further adjusting for the neighborhood deprivation index (quartiles). For face mask-use analysis, compared to individuals who wore face masks none of the time, the adjusted HRs for predicted COVID-19 were 0.27 (95% CI 0.19–0.39) for individuals who wore face masks sometimes, 0.35 (95% CI 0.28–0.43) for individuals who wore face masks most of the time, and 0.36 (95% CI 0.30–0.44) for individuals who wore face masks always (*P*_linear-trend_ < 0.001) after further adjusting for the neighborhood deprivation index (quartiles).

### Risk of predicted COVID-19 with social distancing and face mask use using inverse probability weighting (IPW)

To investigate the generalizability of our results, we conducted inverse probability weighting (IPW) analyses to examine whether correction for age, sex, race, and ethnicity-based demographic differences changes our main finding for social distancing and face mask use. In IPW analyses, we observed a similar association of social distancing and the slightly stronger association of face mask use with the risk of predicted COVID-19. Compared to participants living in communities with overall poor social distancing (Grade = F), the adjusted HRs for predicted COVID-19 at 14 days were 0.82 (95% CI 0.72–0.94) for fair (Grade = D), 0.78 (95% CI 0.66–0.93) for good (Grade = C), and 0.68 (95% CI 0.51–0.91) for excellent (Grade = A/B) social distancing using IPW (*P*_linear-trend_ = 0.004). Moreover, compared to individuals who wore face masks none of the time, the adjusted HRs for predicted COVID-19 were 0.20 (95% CI 0.13–0.30) for individuals who wore face masks sometimes, 0.31 (95% CI 0.24–0.40) for individuals who wore face masks most of the time, and 0.30 (95% CI 0.25–0.38) for individuals who wore face masks always using IPW (*P*_linear-trend_ < 0.001).

### Quantitative bias analysis

We classified a participant to have ‘Predicted COVID-19’ based on a symptom score based on a stringent threshold which yields high specificity for COVID-19 with a tradeoff for sensitivity. Therefore, we ran ~2000 simulation models to calculate the likely value of the true HR assuming a range of possible sensitivity values from 10 to 100%, and calculated the mean HR assuming that the true proportion of COVID-19 cases is greater than 2% during the follow-up period. We can infer that if the Predicted COVID-19 model has a sensitivity of at least 30%, our finding of a reduced risk of COVID-19 associated with stronger community-level social-distancing measures is likely true (Supplementary Table 8). Also, our estimates of HR = 0.69 are unlikely to be strongly biased away from the null assuming a sensitivity of at least 60%. Thus, our findings using the predicted COVID-19 model may be robust to a possible range of sensitivities (given the high specificity of the threshold that we selected). For mask use, we observed that our findings were robust even if the Predicted COVID-19 model has a sensitivity as low as 10% (Supplementary Table 9).

## Discussion

In this prospective study of 198,077 participants using a real-time mobile phone application in the US, we observed that individuals living in communities with the greatest social distancing had a 31% lower risk of predicted COVID-19 compared with those living in communities with poor social distancing, with maximum benefit evident after a latency period of 14 days. Furthermore, among individuals living in communities with poor social distancing, individuals who reported wearing face masks ‘always’ outside of the home had a 62% reduced risk of predicted COVID-19 compared to individuals who wore face masks none of the time.

Notably, a reduction in average distance traveled and nonessential visitation in the community was associated with a reduced risk of predicted COVID-19. In contrast, close contact as measured by human encounters was not associated with predicted COVID-19. This suggests that average distance traveled and nonessential visitation, as measures of independent mobility, may be more reflective of effective social distancing than measures based on assessing proximity between two devices. It is also possible that the criterion to define human encounters based on devices <50 meters apart may not be optimal to study COVID-19 transmission. In subgroup analysis, we did not observe the inverse associations between living in communities with the greater social distancing and risk of COVID-19 among individuals aged greater than 55 years, having health problems requiring stay-at-home, and regularly using mobility aids. For those individuals, living in a community with the greatest social distancing may not play an important role in reducing COVID-19 risk due to their limited mobility and a lower likelihood of social interaction in crowded spaces. Noticeably, the inverse association between living in a community with greater social distancing and the risk of predicted COVID-19 was most consistently observed among younger individuals without significant health problems or limitations in mobility.

We observed that the disease burden of COVID-19 at the start of the social-distancing measurement did not influence the association of social distancing and personal use of a face mask with the risk of predicted COVID-19. We also observed that the association of social distancing with reduced risk of predicted COVID-19 was present both in areas where the epidemic was slowing or maintained (Rt ≤ 1.0) as well as in areas where COVID-19 was actively spreading (Rt > 1.0). We similarly observed that the benefit of personal use of a face mask was observed in regions and time periods in which there was epidemic slowing/maintenance or growth. These findings imply that baseline risk did not impact the relative benefits of social-distancing policies and/or face mask use.

In our study, we used predicted COVID-19 as a proxy for a positive COVID-19 test due to the small number of COVID-19 test-positive app users during the study period. The small fraction of positive COVID-19 tests among all participants (0.31%) may be largely influenced by the limited availability of COVID-19 testing during the study period. A recent study demonstrated that more than 80% of individuals with a COVID-19 infection in the US went undetected in March 2020^29^. Moreover, another study in 10 sites across the US reported that the estimated number of COVID-19 infections was 6–24 times greater per site than the number reported from March 23 to May 12^30^. Therefore, the association between the social distancing observed within one’s community and a positive COVID-19 test should be further investigated in studies in which there was a higher prevalence of testing.

Our findings are consistent with previous ecological studies investigating the effect of social distancing on risk of COVID-19^11–18^. In one recent study that also used estimates of social distancing based on Unacast data, each one-unit increase in social distancing was associated with a 26% reduced risk of COVID-19 incidence and a 31% reduced risk of COVID-19 mortality^12^ at the county level. In a separate study, COVID-19 epidemic case growth rates declined by ~1% per day beginning four days after statewide social-distancing measures were implemented^11^. In addition, estimated rates of COVID-19 cases were increased in border counties in Iowa which did not issue a stay-at-home order compared with border counties in Illinois which did issue a stay-at-home order^13^. Another study based on 149 countries demonstrated that any physical distancing intervention was associated with a 13% reduced risk of COVID-19 incidence^31^. These findings add to this body of evidence as we estimate the impact of social distancing in the community on individual-level outcomes.

Other studies have shown that face mask use is associated with a lower risk of COVID-19 on a population scale^8,15,19–28,32–34^. Particularly, three previous studies investigating the effect of self-reported face mask use on the risk of COVID-19 demonstrated the ORs (odds ratio) from 0.21 to 0.30, which were consistent with our finding (0.36 HR for always use)^24–26^. In one recent study among healthcare workers, universal face mask use was associated with a lower rate of COVID-19 in a hospital setting^27,35^. A recent meta-analysis demonstrated that face mask use was associated with a 85% reduced risk of viral infection causing COVID-19, SARS (severe acute respiratory syndrome), or MERS (Middle East respiratory syndrome)^8^. While the role of a face mask in protecting other individuals is well-recognized, we observed that a face mask may also protect individuals who wear them, as has been described by others^33^.

This study has several strengths. First, we used a mobile application to rapidly collect prospective data from a large population on known or suspected COVID-19 personal risk factors, such as face mask use. This is a significant advantage over existing studies which cannot concurrently examine the impact of personal interventions to reduce exposure risk with community-scale data. Second, we collected data from participants initially free of a positive COVID-19 test and any symptoms, which allowed a prospective assessment of incident symptoms with minimal recall or collider bias^36,37^, or reverse causality. Third, we assessed COVID-19 incidence according to a validated symptom assessment which minimizes the biases associated with geographic variation in access^38^ to COVID-19 testing on estimates of COVID-19 incidence, which may bias effect estimates away from or towards the null (e.g., social distancing associated with reduced test access or increased test-seeking behavior). This also allows us to better assess the impact of social distancing on COVID-19 according to different latency periods since it minimizes the time delay between onset of infection, obtaining a test, and reporting of the result, which has been estimated to be delayed by as long as a week in some areas of the US.^39,40^. Last, our findings emphasizing the efficacy of social distancing and the face mask use to reduce the risk of COVID-19 is relevant to many other settings, including other countries for which additional risk mitigation strategies, such as mass vaccination, remain unattainable in the near term.

There are several limitations to our study. First, our information on risk factors and symptoms are collected by self-report. Although information based on clinical records and testing would be more accurate, given the rapid pace of the pandemic and the limited availability of medical care and testing, self-reported information is more feasible to collect longitudinally and prospectively among a large number of participants and minimizes recall bias or selection bias (e.g., preferentially capturing severe cases through hospitalization records or death reports). Second, since our cohort is not a random sampling of the population, there remains a possibility for selection or collider bias^36,37^, reverse causality, or generalizability. We acknowledge the potential of reverse causality, such as COVID-19 symptoms leading to behavior changes, including social distancing or face mask use. Moreover, we acknowledge the potential of collider bias since our study relies on voluntary participation which may lead to a greater likelihood of participants with COVID-19 symptoms or those more likely to observe social distancing or face mask use to provide data. To minimize these potential biases, we conducted prospective analyses after excluding participants who had any symptoms related to COVID-19 or who had tested positive for COVID-19 prior to the start of follow-up. We also acknowledge that data collection through smartphone adoption has comparatively lower penetrance among certain socioeconomic groups and that participants of an app study may have a differential likelihood of reporting symptoms^41^. Third, it is possible that the personal risk factors for COVID-19 that we assessed here, such as wearing a face mask, may be confounded by other behaviors, such as hand washing, that reduce infection risk. Since the app did not collect the data regarding the other behaviors, we were not able to adjust for them. However, there is growing evidence that COVID-19 may spread through aerosols^42–44^. Since hand washing does not effectively prevent aerosol transmission while the face mask use does^45^, it is less likely that our findings were confounded by hand washing. Fourth, the social-distancing metrics used as an exposure are not reflective of actual user mobility. There may be non-differential misclassification of exposure status by region if county-level factors are correlated with the individual-level heterogeneity of each mobility metric (e.g., younger app users in an urban area with high mobility). Fifth, our analysis was focused on symptomatic COVID-19. However, it is likely that an association between social distancing and face mask use with the risk of asymptomatic spread would be similar. Sixth, while personal face mask use and other covariates were based on individual-level data reported through the app, the social-distancing measures are based on regionally aggregated data assigned to each app user. Last, we were not able to collect additional information on the specific settings of the face mask use (e.g., indoor vs outdoor) due to space limitations on the app and to minimize participant burden.

In conclusion, within a large population-based sample of individuals in the US, we demonstrated a significantly reduced risk of predicted COVID-19 infection among individuals living in communities with a greater social-distancing grade at 14 days either in regions or time periods experiencing either epidemic slowing or growth. Among participants who lived in a community with poor social distancing, wearing a face mask was associated with reduced risk. These findings provide additional support for the efficacy of nonpharmaceutical interventions in reducing COVID-19 incidence and spread and suggest that the benefits of such interventions will become most evident at 14 days after implementation. Despite the advent of several highly effective and safe vaccines, it remains unclear as to when herd immunity will be achieved, particularly in lower-income countries. Thus, social distancing and mask-wearing remain critically important near-term strategies to limit the spread of COVID-19.

## Methods

### Study population

Our study population includes all participants enrolled in the COVID Symptom Study smartphone application (“app”) from March 29, 2020 to July 16, 2020 in the US. The app is a freely available program developed by Zoe Ltd. in collaboration with researchers and clinicians at Massachusetts General Hospital and King’s College London. The data were collected using the app in the US, the UK, and Sweden. However, we restricted our study population to the US because social-distancing data provided by Unacast was only available in the US. Participants using this app reported demographic information and comorbidities at baseline and were encouraged to report on their current health condition daily to allow for the longitudinal, prospective collection of symptoms and COVID-19 testing results^46^. Participants were recruited through general and social media outreach, as well as direct invitations from the investigators of long-running prospective cohorts to study participants^47^. At enrollment, participants provided informed consent to the use of aggregated information for research purposes and agreed to applicable privacy policies and terms of use. This research study was approved by the Partners Human Research Committee (Institutional Review Board Protocol 2020P000909). This protocol is registered with ClinicalTrials.gov (NCT04331509).

### Assessment of predicted COVID-19 and personal risk factors

Upon first use of the app, participants were asked to provide baseline demographic factors, including their zip code of residence, and answered separate questions about suspected risk factors for COVID-19 (Table 1)^46^. On first use and upon daily reminders, participants were asked if they felt physically normal, and if not, their symptoms, including fever, persistent cough, fatigue, loss of smell/taste, and diarrhea, among others^46^. Participants were also asked if they had been tested for COVID-19, and if yes, the results (none, negative, waiting, or positive). To validate the self-reported diagnosis, a subset of individuals who had reported that they had been tested for COVID-19 in the CSS app were invited to provide a copy of COVID-19 test results. A review was conducted by independent reviewers who were blinded to their original self-report responses. Among 235 participants, self-reported COVID-19 testing demonstrated a positive predictive value of 77% and a negative predictive value of 97% for confirmed medical record results. The population density was calculated from Census data for all Zip Code Tabulation Areas (ZCTA) in the US. For socioeconomic status, we calculated Neighborhood Deprivation Index (NDI) using principal components analysis^48^. More specifically, we identified a total of twenty-five variables to assess. The variables included twenty variables identified by the previous study^48^, in addition to another five variables that we identified from the literature as indicators of neighborhood-level deprivation (median household income in thousands, percent insured, average household size, population density per square mile, and percent of nonessential workers). We used principal component analysis to calculate the standardized first principal component. Particularly, we retained the variable if the variable had a loading above 0.25, and the lower 95% confidence limit of the variable loading is not below the lower 95% confidence limit for the median variable loading. Based on these criteria, we retained seven variables for the NDI (percent males in management, percent females in management, percent males in professional occupations, percent females in professional occupations, the median household value in thousands, percent males and females with more than a bachelor education, and percent of nonessential workers). The daily estimated effective reproductive number (Rt), the average number of secondary cases arising from a single case for a given day in each state, was extracted from rt.live, which was provided the case data from the COVID Tracking Project^32,49^. Rt then dichotomized as epidemic slowing/maintenance period (Rt ≤ 1) or epidemic growth period (Rt > 1) for Rt analyses. Because a report of a positive COVID-19 test depends on access to testing and incorporates a variable delay between symptoms and testing, we used a previously published symptom-based classifier that predicts COVID-19 (Predicted COVID-19) as our primary outcome measure^50^. Between March 24 and April 21, 2020, 2,450,569 UK and 168,293 US individuals enrolled in the COVID Symptom Study smartphone application reported symptoms, and 6452 UK and 726 US individuals reported a positive COVID-19 test. To build a prediction model, the UK participants were randomly divided into a training set and a test set (ratio: 80:20). Based on the training set, a logistic model generated to predict symptomatic COVID-19 was: Log odds (Predicted COVID-19) = −1.32 − (0.01 × age) + (0.44 × male sex) + (1.75 × loss of smell or taste) + (0.31 × severe or significant persistent cough) + (0.49 × severe fatigue) + (0.39 x skipped meals). The prediction model achieved a sensitivity of 0.65 (95% CI 0.62–0.67) and specificity of 0.78 (95% CI 0.76–0.80) in the test set. In additional validation in the US participants, the prediction model achieved a sensitivity of 0.66 (95% CI 0.62–0.69) and specificity of 0.83 (95% CI 0.82–0.85). Moreover, to further validate our model of predicted COVID-19 based on self-reported symptoms, we conducted a supplementary analysis to estimate the accuracy of this prediction model in relation to COVID-19 test results. We used independent samples from three different countries (US, UK, and Sweden) including participants who joined the app between April 22 and May 31, after the original prediction models were created (among test results from March 24 to April 21). Using a total of 4669 total test results, including 573 positive test results, we found the AUC of >70% in all three countries No evidence of heterogeneity was observed between the AUCs in the three countries (Supplementary Fig. 1). We used testing positive for COVID-19 as our secondary outcome measure. To examine the influence of COVID-19 incidence on our results, we included the daily county-level test-positive COVID-19 incidence estimated by the Center for Systems Science and Engineering at Johns Hopkins University as a covariate^51,52^.

### Assessment of community social distancing and personal face mask use

We assigned each individual participant a social-distancing grade within their communities based on their zip code of residence. We used data provided by Unacast^53^ that estimated county-level social distancing for each calendar day according to the smartphone-based GPS activity of all devices assigned to their longest recorded location. Compared to the same day of the week during the pre-COVID-19 period (defined by Unacast as the four weeks prior to March 8, 2020), Unacast estimated, for each day, the percent reduction in each of the three metrics—metric 1, the average distance traveled per device; metric 2, nonessential visitation (e.g., restaurants, department stores, hair salons); and metric 3, human encounters calculated as two devices in close proximity (i.e., spatial distance of ≤50 m and temporal distance of ≤60 min)^53^. Unacast assigned grades (A, B, C, D, and F) using predefined cutoff points for each metric and calculated an overall social-distancing grade (Supplementary Methods), with grade A indicating the greatest social distancing and F the poorest social distancing. For all analyses, we combined grades A and B due to a limited number of individuals living in counties assigned to grade A. For personal face mask use, we used the individual-level information collected through the app. Beginning on June 12, 2020, app users received supplementary questions regarding face mask use based on the query “In the last week, did you wear a face mask when outside the house?”. The answer was collected according to the frequency of face mask use (none of the time, sometimes, most of the time, or always) and updated every time when the app users log into the app by asking the face mask use in the last week.

### Statistical analysis

We conducted prospective analyses after excluding participants who had any symptom related to COVID-19 or who had tested positive for COVID-19 prior to start of follow-up to minimize reverse causality and collider bias^36,37^. Follow-up time started when participants first reported on the app and accrued until they developed predicted COVID-19, or the time of last data entry prior to July 16, whichever occurred first. We used updated, time-varying community social-distancing exposure data as our primary independent variable. Community-level social-distancing exposure data and corresponding follow-up time was mapped to each individual and updated each time they logged in the app to provide updated symptom information. We also used time-varying face mask-use exposure data for the association between self-reported personal use of masks and predicted COVID-19. Cox proportional hazards regression models stratified by age, state, and calendar date at study entry were used to calculate unadjusted and adjusted hazard ratios (HRs) and 95% confidence intervals (CIs) of predicted COVID-19. Covariates were selected a priori based on putative risk factors and included race (White, Black, Asian, other race), sex (male, female), population density (quartiles), current smoking, work as a frontline healthcare worker, interaction with suspected or documented COVID-19, and history of diabetes, heart disease, lung disease, kidney disease (each yes/no), and neighborhood deprivation index (quartiles). Missing data for categorical variables were included as a missing indicator.

To minimize any variation of estimated daily social-distancing grade associated with the day of the week (e.g., Sunday vs. Monday), we used a seven-day average of community social-distancing grade as the exposure for each participant. To access the incubation period, we first examined the latency between community social-distancing grade and predicted COVID-19 using varying lag times (0 day, 7 days, 14 days, 21 days, and 28 days). For example, for a latency of 7 days, we used social-distancing grade exposure on April 1 for predicted COVID-19 outcome measures on April 8, grade on April 2 for follow-up on April 9, and so forth (Supplementary Fig. 2). For subgroup analysis according to daily state-level Rt, we used a 21-day latency since this corresponded to the start of the seven-day average social-distancing exposure with a 14-day latency. Two-sided *P* values of <0.05 were considered statistically significant for main analyses. All statistical analyses were performed using R software, version 3.6.1 (R Foundation).

### Reporting summary

Further information on research design is available in the Nature Research Reporting Summary linked to this article.

## Supplementary information

Supplementary Information

Reporting Summary

## Data Availability

Data collected in the app are being shared with other health researchers through the NHS-funded Health Data Research UK (HDRUK)/SAIL consortium, housed in the UK Secure e-Research Platform (UKSeRP) in Swansea. Anonymized data collected by the symptom tracker app can be shared with bonafide researchers via HDRUK, provided the request is made according to their protocols and is in the public interest (see https://web.www.healthdatagateway.org/dataset/fddcb382-3051-4394-8436-b92295f14259/). US investigators are encouraged to coordinate data requests through the COPE Consortium (www.monganinstitute.org/cope-consortium). Data updates can be found at https://covid.joinzoe.com.

## Code availability

Code for data extraction is available at https://github.com/KCL-BMEIS/ExeTera/.
