# Supplementary material for: Association of social distancing and face mask use with risk of COVID-19
Source: Nat Commun. 2021 Jun 18;12:3737. doi: 10.1038/s41467-021-24115-7 (PMC8213701; doi:10.1038/s41467-021-24115-7)
Supplement: Supplementary file 1 — Supplementary Information [file 41467_2021_24115_MOESM1_ESM.pdf]

Supplementary Information

Association of social distancing and face mask use with risk of COVID-19

Sohee Kwon[1,2]*, Amit D. Joshi[1,2]*, Chun-Han Lo[1,2], David A. Drew[1,2] , Long H. Nguyen[1,2,3], Chuan-Guo Guo[1,2,4], Wenjie Ma[1,2], Raaj S. Mehta[1,2], Fatma Mohamed Shebl[5], Erica T. Warner[1,6], Christina M. Astley[7,8], Jordi Merino[9,10,11], Benjamin Murray[12], Jonathan Wolf[13], Sebastien Ourselin[12], Claire J. Steves[14], Tim D. Spector[14], Jaime E. Hart[15,16], Mingyang Song[1,2,17,18], Trang VoPham[19,20], Andrew T. Chan[1,2,9,21‡]

*Contributed equally to this work

- Supplementary Methods
- Supplementary Figure 1. Area under the ROC Curve of predicted COVID-19 model in independent samples from three different countries (US, UK and Sweden)
- Supplementary Figure 2. Latency between community social distancing grade and predicted Covid-19 at various time intervals (7, 14, and 21 days latency)
- Supplementary Figure 3. Overall community social distancing grade in the U.S. over time
- Supplementary Table 1. Baseline characteristics of study participants according to frequency of personal face mask use
- Supplementary Table 2. Risk of predicted symptomatic COVID-19 according to overall social distancing grade within subgroups
- Supplementary Table 3. Risk of predicted symptomatic COVID-19 according to frequency of personal face mask use within subgroups
- Supplementary Table 4. Risk of testing positive COVID-19 according to overall social distancing grade
- Supplementary Table 5. Risk of testing positive COVID-19 according to frequency of personal face mask use
- Supplementary Table 6. Risk of predicted COVID-19 according to overall social distancing grade within Rt subgroups
- Supplementary Table 7. Risk of predicted COVID-19 according to frequency of personal face mask use within Rt subgroups
- Supplementary Table 8. Quantitative bias analyses for overall social distancing grade
- Supplementary Table 9. Quantitative bias analyses for face mask use
- Supplementary References

Supplementary Methods

Community social distancing grade

First, Unacast defined pre- and post- COVID19 periods. Pre-COVID19 period was defined as 4 weeks before the 8th of March 2020. Post-COVID19 period is defined as after the 8th of March 2020. Next, Unacast defined baselines for metrics 1,2, and 3: 1) average distance traveled per device; 2) non-essential visitation (e.g., restaurants, department stores, hair salons); and 3) human encounters calculated as two devices in close proximity (i.e., spatial distance of ≤50 m and temporal distance of ≤60 minutes). For metrics 1 and 2, a baseline for each post-COVID19 weekday is the average of the metric on 4 corresponding pre-COVID19 weekdays. For example, for the 30th of March, Monday, the baseline is the average of daily metrics on 4 pre-COVID19 Mondays: 10, 17, 24th of February and 2nd of March. For metric 3, a baseline is the national average of the metric taken over 4 weeks that immediately precede Covid-19 outbreak on March 8th, i.e, Feb 10, 2020 - Mar 8, 2020. After defining baseline values for each metric as continuous variables, Unacast calculated percent reduction in each of these metrics during the post-COVID19 period with respect to the pre-COVID19 values. Thus, the total distance traveled on each post-COVID 19 day compared to a corresponding pre-COVID 19 baseline for metric 1. Similarly, they obtained percent reduction in non-essential visitation for metric 2 and percent reduction in human encounters for metric 3.

Community social distancing was graded by Unacast for each metric 1, 2, and 3 and overall grade assigned as A, B, C, D, and F [1]. For metric 1, percent reduction in average distance traveled per device are categorized and graded as A: >70% decrease, B: 55-70% decrease, C: 40-55% decrease, D: 25-40% decrease, and F: <25% decrease or increase. For metric 2, percent reduction in non-essential visitation are categorized and graded as A: >70% decrease, B: 65-70% decrease, C: 60-65% decrease, D: 55-60% decrease, and F: <55% decrease or increase. For metric 3, percent reduction in human encounters are categorized and graded as A: >94% decrease, B: 94-82% decrease, C: 82-74% decrease, D: 74-40% decrease, and F: <40% decrease or increase. The overall grade was calculated based on metric 1, metric 2, and metric 3 as the average between the three numeric grades by Unacast.

For metric 2, non-essential visitation include (but are not limited to): restaurants (multiple kinds), department and clothing stores, jewelers, consumer electronics stores, cinemas and theaters, office supply stores, spas and hair salons, gyms and fitness/recreation facilities, car dealerships, hotels, craft, toy, and hobby shops.

Social distancing data was provided at the county-level. Data are not available for counties with a population less than 1,000; where less than 100 smartphone devices were observed for 70% of the days during the pre-COVID-19 period; or where less than 5 non-essential venues or 100 non-essential venue visits occurred during the pre-COVID-period.

Community-level daily social distancing grades were assigned to each individual in the app using Zip code and the time they log in. Unacast provided social distancing grades with Federal Information Processing Standards (FIPS) county code, a five-digit code which uniquely identified counties and county equivalents in the US. We matched the FIPS to Zip code and then assigned the daily social distancing grades using Zip code and the time they log in.

Supplementary Figure 1. Area under the ROC Curve of predicted COVID-19 model in independent samples from three different countries (US, UK and Sweden)

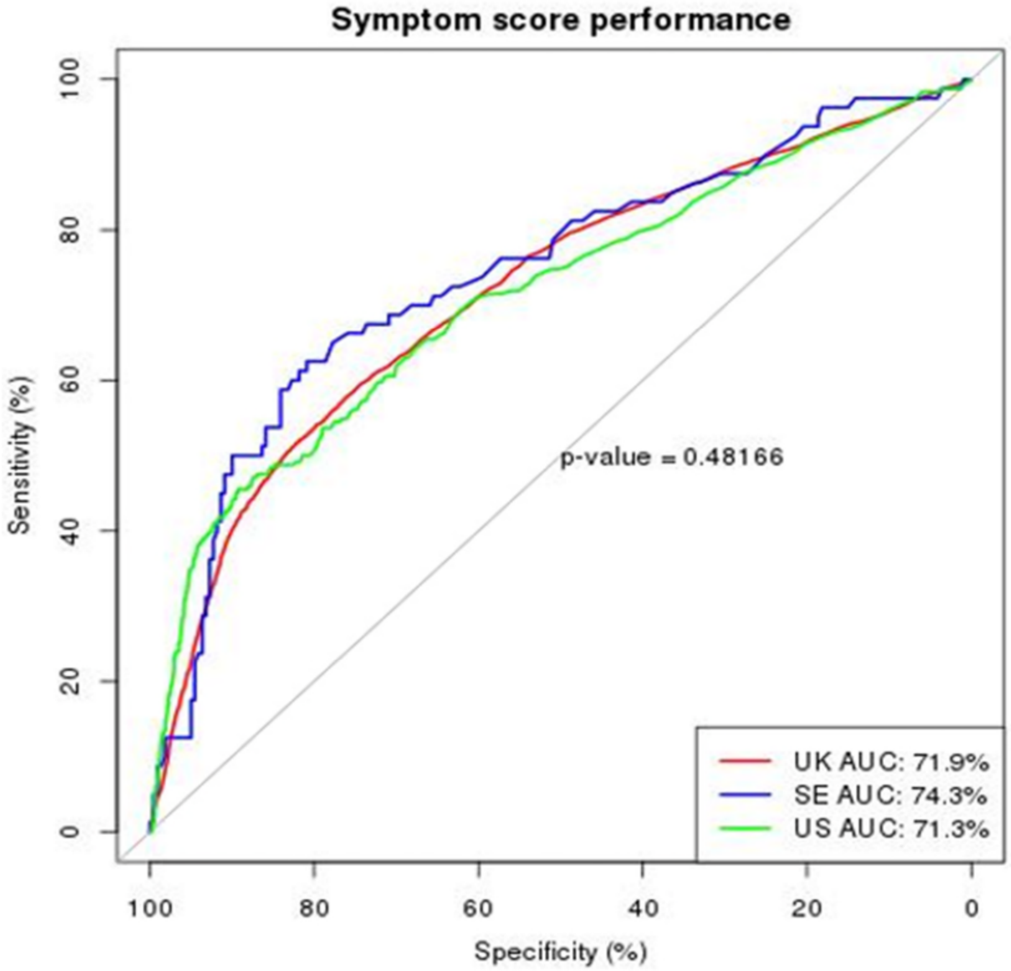

Abbreviations: AUC (Area under the ROC Curve), UK (United Kingdom), SE (Sweden), US (United States**)**

Supplementary Figure 2. Latency between community social distancing grade exposure and predicted Covid-19 outcome at various time intervals (7, 14, and 21 days latency)

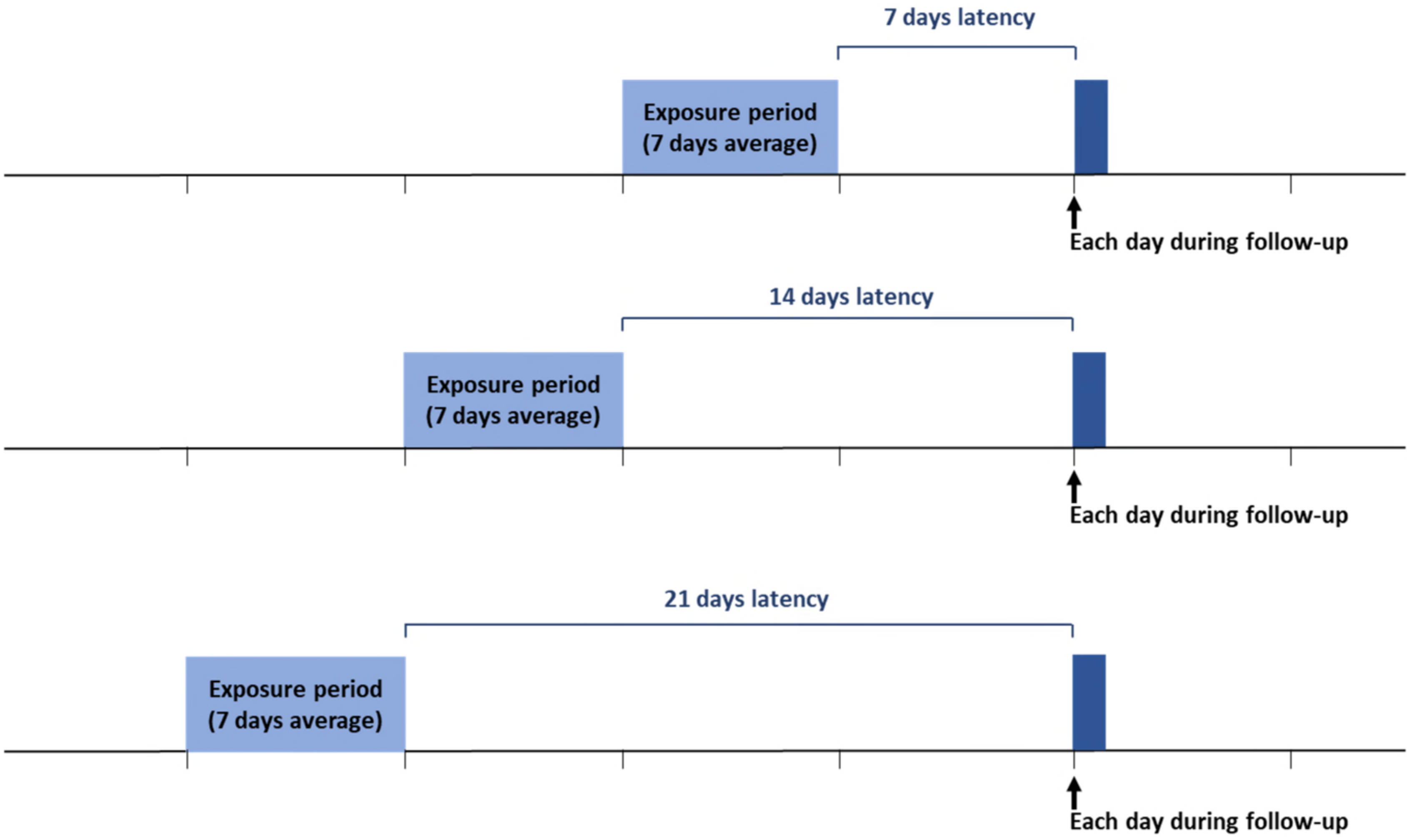

Supplementary Figure 3. Overall community social distancing grade in the U.S. [a] over time

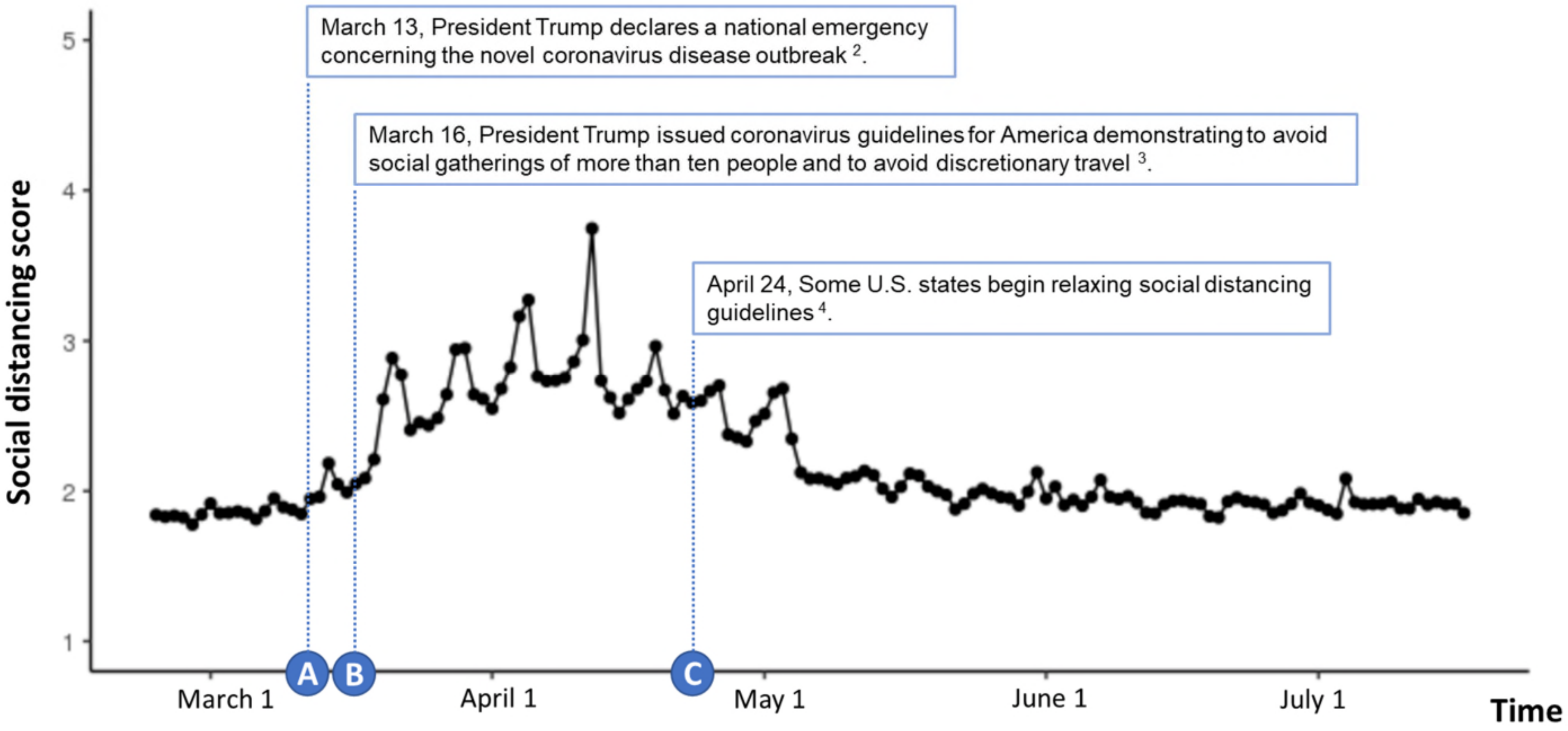

[a] Social distancing score is averaged across the counties in U.S. Excellent (A/B); social distancing score is greater than 4. Good (C); social distancing score is greater than 2.5 and less than 4. Fair (B); social distancing score is greater than 1.5 and less than 2.5. Poor (F); social distancing score is less than 1.5.

Supplementary Table 1. Baseline characteristics of study participants according to frequency of personal face mask use [a]

		Overall n = 134597	None of the time n = 76911	Sometimes n = 7568	Most of the time n = 18074	Always n = 32044
Age (years), %						
	<25	5.8	5.1	9.7	6.9	6.0
	25-34	7.2	5.7	7.8	10.4	8.9
	35-44	11.5	9.6	14.3	14.8	13.5
	45-54	13.8	12.4	15.4	16.2	15.3
	55-64	21.4	22.0	20.9	20.9	20.5
	≥65	40.4	45.4	32.0	30.9	35.8
	Missing	0.0	0.0	0.0	0.0	0.0
Male sex, %		35.3	35.7	41.8	36.1	32.3
Race/Ethnicity [b], %						
	White, non-Hispanic	82.5	82.8	86.9	85.4	79.3
	Hispanic/Latinx	4.3	3.7	3.6	4.1	6.3
	Black	2.5	2.4	1.2	1.6	3.6
	Asian	3.4	3.4	1.8	2.6	4.3
	Mixed/Other race	2.5	2.3	2.3	2.6	2.9
	Prefer not to say	1	1.0	0.9	1.0	1.0
	Missing	3.7	4.4	3.3	2.7	2.7
Current smoker, %		3.9	3.5	5.0	4.1	4.5
	Missing	0.0	0.0	0.0	0.0	0.0
Comorbidities, %						
	Diabetes	5.9	6.6	4.0	3.9	5.8
	Heart Disease	6.9	7.4	5.3	5.4	6.7
	Lung Disease	11	11.6	8.6	9.4	10.9
	Kidney Disease	1.6	1.7	1.1	1.2	1.7
Population density, %						
	Quartile 1	24.5	25.1	29.1	24.1	22.3
	Quartile 2	24.6	25.0	25.2	24.0	24.1
	Quartile 3	25.1	24.7	24.3	26.6	25.4
	Quartile 4	25.2	24.7	20.9	24.9	27.6
	Missing	0.5	0.6	0.5	0.4	0.6
Frontline healthcare worker, %		7.9	6.9	7.5	9.4	9.4
Interaction with suspected or documented Covid-19, %		7.9	6.6	8.3	9.8	9.8
Health problems requiring stay-at-home [c], %		4.4	4.4	2.9	3.0	5.4
Regular use mobility aid [d], %		2.0	2.3	1.1	1.1	2.2
Health problems limiting activities [e], %		8.4	8.6	5.7	6.6	9.6

Neighborhood Deprivation Index, %					
Quartile 1	26.8	26.5	24.5	28.5	27.1
Quartile 2	23.9	24.0	23.7	24.1	23.5
Quartile 3	24.2	24.2	24.3	23.2	24.6
Quartile 4	24.5	24.6	26.8	23.6	24.1
Missing	0.7	0.7	0.7	0.7	0.8

[a] The frequency of personal face mask use was collected from 139,690 participants beginning on June 12, 2020 based on the query "In the last week, did you wear a face mask when outside the house?".

[b] The proportion of race was calculated among the participants who received the race question which was added at April 18, 2020.

[c] Asked as "In general, do you have any health problems that require you to stay at home?"

[d] Asked as "Do you regularly use a stick, walking frame or wheelchair to get about?"

[e] Asked as "In general, do you have any health problems that require you to limit your activities?"

Supplementary Table 2. Risk of predicted COVID-19 according to overall social distancing grade within subgroups [a]

Variables		No. of Case	Adjusted HR (95% CI) [b]		P value for Interaction
			Poor (F)	Excellent (A/B)	
Age, years					
	≤ 35	619	1 [Reference]	0.73 (0.41-1.29)	0.001
	35-55	688	1 [Reference]	0.47 (0.26-0.84)	
	> 55	372	1 [Reference]	1.08 (0.60-1.95)	
Race					
	White, non-Hispanic	1228	1 [Reference]	0.60 (0.32-1.12)	0.40
	Black/Hispanic	170	1 [Reference]	0.81 (0.35-1.91)	
	Asian/Others	113	1 [Reference]	0.93 (0.37-2.38)	
Sex					
	Male	801	1 [Reference]	0.60 (0.33-1.09)	0.53
	Female	878	1 [Reference]	0.53 (0.29-0.97)	
Population density					
	≤ median	806	1 [Reference]	0.48 (0.27-0.87)	0.004
	> median	860	1 [Reference]	0.89 (0.47-1.69)	
Interaction with suspected or documented Covid-19					
	Yes	272	1 [Reference]	0.51 (0.25-1.06)	0.65
	No	1405	1 [Reference]	0.58 (0.33-1.02)	
Health problems requiring stay-at-home [c]					
	Yes	138	1 [Reference]	1.18 (0.54-2.57)	0.02
	No	1541	1 [Reference]	0.53 (0.30-0.94)	
Regular use mobility aid [d]					
	Yes	48	1 [Reference]	2.13 (0.83-5.48)	0.002
	No	1631	1 [Reference]	0.55 (0.31-0.97)	
Health problems limiting activities [e]					
	Yes	250	1 [Reference]	0.71 (0.35-1.44)	0.27
	No	1429	1 [Reference]	0.53 (0.29-0.95)	

Abbreviations: HR (hazard ratio), CI (confidence interval)

Cox proportional hazards regression models were used to calculate adjusted HRs and 95% CIs.

[a] Day-14 is applied for models.

[b] Adjusted models are adjusted for the same covariates as the model 2 in Table 2.

[c] Asked as “In general, do you have any health problems that require you to stay at home?”

[d] Asked as “Do you regularly use a stick, walking frame or wheelchair to get about?”

[e] Asked as “In general, do you have any health problems that require you to limit your activities?”

Supplementary Table 3. Risk of predicted COVID-19 according to frequency of personal face mask use within subgroups

Variables	No. of Case	Adjusted HR (95% CI) [a]		P value for Interaction
		None of the time	Always	
Age, years				
≤ 35	460	1 [Reference]	0.34 (0.27-0.42)	0.049
35-55	501	1 [Reference]	0.33 (0.27-0.41)	
> 55	268	1 [Reference]	0.49 (0.37-0.66)	
Race				
White, non-Hispanic	957	1 [Reference]	0.35 (0.29-0.41)	0.88
Black/Hispanic	139	1 [Reference]	0.33 (0.22-0.48)	
Asian/Others	90	1 [Reference]	0.38 (0.24-0.62)	
Sex				
Male	621	1 [Reference]	0.35 (0.28-0.42)	0.97
Female	607	1 [Reference]	0.34 (0.28-0.42)	
Population density				
≤ median	539	1 [Reference]	0.32 (0.26-0.40)	0.35
> median	649	1 [Reference]	0.37 (0.30-0.44)	
Interaction with suspected or documented Covid-19				
Yes	198	1 [Reference]	0.27 (0.19-0.38)	0.11
No	1029	1 [Reference]	0.36 (0.31-0.43)	
Health problems requiring stay-at-home [b]				
Yes	94	1 [Reference]	0.33 (0.20-0.54)	0.87
No	1133	1 [Reference]	0.35 (0.30-0.41)	
Regular use mobility aid [c]				
Yes	30	1 [Reference]	0.19 (0.06-0.55)	0.22
No	1197	1 [Reference]	0.35 (0.30-0.41)	
Health problems limiting activities [d]				
Yes	163	1 [Reference]	0.28 (0.19-0.41)	0.24
No	1064	1 [Reference]	0.36 (0.30-0.42)	

Abbreviations: HR (hazard ratio), CI (confidence interval)

Cox proportional hazards regression models were used to calculate adjusted HRs and 95% CIs.

[a] Adjusted models are adjusted for the same covariates as the model 2 in Table 2.

[b] Asked as “In general, do you have any health problems that require you to stay at home?”

[c] Asked as “Do you regularly use a stick, walking frame or wheelchair to get about?”

[d] Asked as “In general, do you have any health problems that require you to limit your activities?”

Supplementary Table 4. Risk of testing positive COVID-19 according to overall social distancing grade

Overall social distance grade [a]	Poor (F)	Fair (D)	Good (C)	Excellent (A/B)	P value for Trend [b]
No. of Case/ Person-time (days)	188 / 5,050,742	145 / 3,981,230	262 / 2,974,430	20 / 366,546	
Model 1 HR (95% CI) [c]	1	0.90 (0.63-1.27)	1.14 (0.77-1.68)	0.77 (0.42-1.41)	0.66
Model 2 HR (95% CI) [d]	1	0.97 (0.68-1.39)	1.08 (0.72-1.63)	0.75 (0.40-1.41)	0.94

Abbreviations: HR (hazard ratio), CI (confidence interval)

Cox proportional hazards regression models were used to calculate HRs and 95% CIs.

[a] Overall social distancing grades are denoted as Poor (F grade), Fair (D grade), Good (C grade), and Excellent (A+B grade). Overall social grade categories (A, B, C, D, and F) are provided by Unacast.

[b] Two-sided P values for trend were calculated using the median value of each category as a continuous variable.

[c] Model 1 was stratified by age (<25, 25-34, 35-44, 45-54, 55-64, or ≥65), state, and calendar date at study entry.

[d] Model 2 was stratified by age (<25, 25-34, 35-44, 45-54, 55-64, ≥65), state, and calendar date at study entry and further adjusted for race (white, black, Asian, or other), sex (male or female), population density of residence (quartiles), current smoking, frontline healthcare worker, interaction with suspected or documented Covid-19, history of diabetes, heart disease, lung disease, and kidney disease (each yes or no).

Supplementary Table 5. Risk of testing positive for COVID-19 according to frequency of personal face mask use

		Frequency of personal face mask use[a]				
		None of the time	Sometimes	Most of the time	Always	P for trend [b]
Overall social distancing						
	No. of Case/ Person-time (days)	103 / 2,653,287	6 / 217,409	17 / 587,533	50 / 1,106,035	
	Model 1 HR (95% CI) [c]	1 [Reference]	0.25 (0.10-0.59)	0.31 (0.18-0.55)	0.40 (0.26-0.62)	2.37×10^{-5}
	Model 2 HR (95% CI) [d]	1 [Reference]	0.24 (0.10-0.58)	0.30 (0.17-0.53)	0.37 (0.24-0.57)	6.50×10^{-6}
Poor (F) [e]						
	No. of Case/ Person-time (days)	87 / 1,975,027	5 / 163,974	12 / 447,336	45 / 837,547	
	Model 1 HR (95% CI)	1 [Reference]	0.26 (0.10-0.67)	0.25 (0.13-0.47)	0.38 (0.24-0.60)	2.31×10^{-5}
	Model 2 HR (95% CI)	1 [Reference]	0.25 (0.10-0.66)	0.23 (0.11-0.44)	0.34 (0.21-0.55)	6.84×10^{-6}

Abbreviations: HR (hazard ratio), CI (confidence interval)

Cox proportional hazards regression models were used to calculate adjusted HRs and 95% CIs.

[a] The frequency of personal face mask use was collected from 139,690 participants beginning on June 12, 2020 based on the query “In the last week, did you wear a face mask when outside the house?”.

[b] Two-sided P values for trend were calculated as an ordinal variable.

[c] Model 1 was stratified by age (<25, 25-34, 35-44, 45-54, 55-64, or ≥65), state, and calendar date at study entry.

[d] Model 2 was stratified by age (<25, 25-34, 35-44, 45-54, 55-64, ≥65), state, and calendar date at study entry and further adjusted for race (white, black, Asian, or other), sex (male or female), population density (quartiles), current smoking, frontline healthcare worker, interaction with suspected or documented Covid-19, history of diabetes, heart disease, lung disease, and kidney disease (each yes or no).

[e] Only poor(F) social distance grade group is demonstrated due to a limited number of participants in Fair (D grade), Good (C grade), and Excellent (A+B grade).

Supplementary Table 6. Risk of predicted COVID-19 according to overall social distancing grade within Rt subgroups

		Overall social distancing grade [a]				
Effective reproductive number (Rt)		Poor (F)	Fair (D)	Good (C)	Excellent (A/B)	P value for Trend [b]
≤1.0						
	No. of Case/ Person-time (days)	490 / 1,861,758	743 / 2,316,208	783 / 1,962,081	83 / 250,257	
	Model 1 HR (95% CI) [c]	1 [Reference]	0.87 (0.75-1.01)	0.77 (0.64-0.92)	0.61 (0.46-0.82)	$4.52x10^{-4}$
	Model 2 HR (95% CI) [d]	1 [Reference]	0.88 (0.76-1.02)	0.79 (0.66-0.95)	0.63 (0.47-0.85)	0.002
>1.0						
	No. of Case/ Person-time (days)	1048 / 2,796,848	714 / 1,372,343	569 / 778,131	58 / 90,815	
	Model 1 HR (95% CI)	1 [Reference]	0.84 (0.73-0.97)	0.83 (0.69-1.00)	0.78 (0.56-1.08)	0.05
	Model 2 HR (95% CI)	1 [Reference]	0.85 (0.73-0.99)	0.84 (0.70-1.01)	0.84 (0.60-1.16)	0.11

Abbreviations: HR (hazard ratio), CI (confidence interval)

Cox proportional hazards regression models were used to calculate HRs and 95% CIs.

[a] Overall social distancing grades are denoted as Poor (F grade), Fair (D grade), Good (C grade), and Excellent (A+B grade). Overall social grade categories (A, B, C, D, and F) are provided by Unacast.

[b] Two-sided P values for trend were calculated using the median value of each category as a continuous variable.

[c] Model 1 was stratified by age (<25, 25-34, 35-44, 45-54, 55-64, or ≥65), state, and calendar date at study entry.

[d] Model 2 was stratified by age (<25, 25-34, 35-44, 45-54, 55-64, ≥65), state, and calendar date at study entry and further adjusted for race (white, black, Asian, or other), sex (male or female), population density of residence (quartiles), current smoking, frontline healthcare worker, interaction with suspected or documented Covid-19, history of diabetes, heart disease, lung disease, and kidney disease (each yes or no).

Supplementary Table 7. Risk of predicted COVID-19 according to frequency of personal face mask use within Rt subgroups

Effective reproductive number (Rt)		Frequency of personal face mask use [a] None of the time	Sometimes	Most of the time	Always	P for trend [b]
≤1.0						
	No. of Case/ Person-time (days)	201 / 727,128	5 / 60,831	28 / 160,276	51 / 289,393	
	Model 1 HR (95% CI) [c]	1 [Reference]	0.17 (0.07-0.42)	0.39 (0.25-0.61)	0.37 (0.25-0.55)	4.82×10^{-8}
	Model 2 HR (95% CI) [d]	1 [Reference]	0.16 (0.06-0.41)	0.41 (0.26-0.64)	0.38 (0.25-0.55)	1.07×10^{-7}
>1.0						
	No. of Case/ Person-time (days)	612 / 1,761,812	37 / 137,164	87 / 370,474	173 / 702,160	
	Model 1 HR (95% CI)	1 [Reference]	0.32 (0.22-0.47)	0.32 (0.25-0.41)	0.35 (0.28-0.43)	2.09×10^{-27}
	Model 2 HR (95% CI)	1 [Reference]	0.31 (0.21-0.46)	0.33 (0.25-0.42)	0.36 (0.29-0.44)	1.39×10^{-25}

Abbreviations: HR (hazard ratio), CI (confidence interval)

Cox proportional hazards regression models were used to calculate HRs and 95% CIs.

[a] The frequency of personal face mask use was collected from 139,690 participants beginning on June 12, 2020 based on the query “In the last week, did you wear a face mask when outside the house?”.

[b] Two-sided P values for trend were calculated as an ordinal variable.

[c] Model 1 was stratified by age (<25, 25-34, 35-44, 45-54, 55-64, or ≥65), state, and calendar date at study entry.

[d] Model 2 was stratified by age (<25, 25-34, 35-44, 45-54, 55-64, ≥65), state, and calendar date at study entry and further adjusted for race (white, black, Asian, or other), sex (male or female), population density (quartiles), current smoking, frontline healthcare worker, interaction with suspected or documented Covid-19, history of diabetes, heart disease, lung disease, and kidney disease (each yes or no).

Supplementary Table 8. Quantitative bias analysis for overall social distancing grade

		Overall social distancing grade F vs. Grade A+B<br>Mean of True HR (95% CI)		
		Exp (Mean beta)	Exp (2.5th percentile beta)	Exp (97.5th percentile beta)
Sensitivity	10%	0.94	0.84	1.05
	20%	0.91	0.80	1.05
	30%	0.87	0.79	0.99
	40%	0.84	0.75	0.97
	50%	0.83	0.73	0.91
	60%	0.78	0.66	0.90
	70%	0.76	0.64	0.90
	80%	0.73	0.68	0.80
	90%	0.70	0.64	0.76
	100%	0.68	0.64	0.71

Abbreviations: HR (hazard ratio), CI (confidence interval), Exp (Exponential)

Supplementary Table 9. Quantitative bias analysis for face mask use

		Frequency of personal face mask use (None of the time vs. Always) Mean of True HR (95% CI)		
		Exp (Mean beta)	Exp (2.5th percentile beta)	Exp (97.5th percentile beta)
Sensitivity	10%	0.44	0.41	0.49
	20%	0.43	0.39	0.48
	30%	0.42	0.37	0.48
	40%	0.41	0.36	0.46
	50%	0.40	0.35	0.45
	60%	0.39	0.35	0.45
	70%	0.38	0.34	0.42
	80%	0.37	0.33	0.41
	90%	0.36	0.34	0.39
	100%	0.35	0.33	0.37

Abbreviations: HR (hazard ratio), CI (confidence interval), Exp (Exponential)

SUPPLEMENTARY REFERENCES

1. Unacast Social Distancing Dataset. 2020; https://www.unacast.com/data-for-good. Accessed version from 17 July 2020.

2. House W. Proclamation on declaring a national emergency concerning the novel coronavirus disease (COVID-19) outbreak. White House. 2020. (March 13, 2020).

3. House W. Coronavirus Guidelines for America. White House. 2020. (March 16, 2020).

4. Elassar A. This is where each state is during its phased reopening. *CNN*. (May 27, 2020). https://www.cnn.com/interactive/2020/us/states-reopen-coronavirus-trnd/
