# Supplementary material for: Association of social distancing and face mask use with risk of COVID-19
Source: Nat Commun. 2021 Jun 18;12:3737. doi: 10.1038/s41467-021-24115-7 (PMC8213701; doi:10.1038/s41467-021-24115-7)
Supplement: Supplementary file 2 — Reporting Summary [file 41467_2021_24115_MOESM2_ESM.pdf]

nature research

Corresponding author(s): Andrew T. Chan

Last updated by author(s): May 25, 2021

Reporting Summary

Nature Research wishes to improve the reproducibility of the work that we publish. This form provides structure for consistency and transparency in reporting. For further information on Nature Research policies, see our Editorial Policies and the Editorial Policy Checklist.

Statistics

For all statistical analyses, confirm that the following items are present in the figure legend, table legend, main text, or Methods section.

n/a	Confirmed	
☐	☒	The exact sample size (n) for each experimental group/condition, given as a discrete number and unit of measurement
☐	☒	A statement on whether measurements were taken from distinct samples or whether the same sample was measured repeatedly
☐	☒	The statistical test(s) used AND whether they are one- or two-sided<br>*Only common tests should be described solely by name; describe more complex techniques in the Methods section.*
☐	☒	A description of all covariates tested
☐	☒	A description of any assumptions or corrections, such as tests of normality and adjustment for multiple comparisons
☐	☒	A full description of the statistical parameters including central tendency (e.g. means) or other basic estimates (e.g. regression coefficient) AND variation (e.g. standard deviation) or associated estimates of uncertainty (e.g. confidence intervals)
☐	☒	For null hypothesis testing, the test statistic (e.g. F, t, r) with confidence intervals, effect sizes, degrees of freedom and P value noted<br>*Give P values as exact values whenever suitable.*
☒	☐	For Bayesian analysis, information on the choice of priors and Markov chain Monte Carlo settings
☒	☐	For hierarchical and complex designs, identification of the appropriate level for tests and full reporting of outcomes
☐	☒	Estimates of effect sizes (e.g. Cohen's d, Pearson's r), indicating how they were calculated

Our web collection on statistics for biologists contains articles on many of the points above.

Software and code

Policy information about availability of computer code

Data collection	Data was collected using the Covid Symptom Study smartphone application, as retrieved in July 2020. The app is a freely available mobile software developed by Zoe Global Ltd. in collaboration with researchers and clinicians at King's College London and Massachusetts General Hospital. Code for data extraction is available at https://github.com/KCL-BMEIS/ExeTera/.
Data analysis	All statistical analyses were performed using R software, version 3.6.1 (R foundation).

For manuscripts utilizing custom algorithms or software that are central to the research but not yet described in published literature, software must be made available to editors and reviewers. We strongly encourage code deposition in a community repository (e.g. GitHub). See the Nature Research guidelines for submitting code & software for further information.

Data

Policy information about availability of data

All manuscripts must include a data availability statement. This statement should provide the following information, where applicable:

- Accession codes, unique identifiers, or web links for publicly available datasets
- A list of figures that have associated raw data
- A description of any restrictions on data availability

Data collected in the app are being shared with other health researchers through the NHS-funded Health Data Research UK (HDRUK)/ SAIL consortium, housed in the UK Secure e-Research Platform (UKSeRP) in Swansea. Anonymised data can be shared with bonafide researchers via HDRUK, provided the request is made according to their protocols and is in the public interest (see https://healthdatagateway.org/detail/9b604483-9cdc-41b2-b82c-14ee3dd705f6). Data updates can be found at https://covid.joinzoe.com

Field-specific reporting

Please select the one below that is the best fit for your research. If you are not sure, read the appropriate sections before making your selection.

- [x] Life sciences
- [ ] Behavioural & social sciences
- [ ] Ecological, evolutionary & environmental sciences

For a reference copy of the document with all sections, see nature.com/documents/nr-reporting-summary-flat.pdf

Life sciences study design

All studies must disclose on these points even when the disclosure is negative.

Sample size	Our study population includes all participants enrolled in the COVID Symptom Study smartphone application ("app") from March 29, 2020 to July 16, 2020 in the U.S. We enrolled 277,798 participants who provided baseline information.
Data exclusions	We excluded 79,721 individuals who did not live in a county with available Unacast data, reported any symptoms or a positive COVID 19 test at enrollment, had <24 hours of follow-up time or who reported a positive COVID-19 test or symptoms of predicted COVID-19 within 24 hours of enrollment. Since this is a prospective analysis, the exclusion criteria were used to exclude any participants who already had the COVID at or prior to baseline and thus would contribute 0 follow-up time. Moreover, excluding participants with COVID-19 prior to start of follow-up would minimize reverse causality and collider bias.
Replication	This is an observational study using unique data resource from the COVID Symptom Study app data and social distancing Unacast data (U.S. only). It could be replicated if similar data with longitudinal follow-up and baseline questionnaires as the COVID Symptom Study app data are available, and if the structure permits prospective analyses.
Randomization	Our primary exposures (social distancing grades and mask-wearing) were not randomized to individuals. Social distancing guidelines and mask use were mandated and/ or enforced by local governments. We tried to control for relevant covariates using stratified analyses and by use of multivariable models.
Blinding	Social distancing and mask wearing behaviors were either self-selected or locally mandated and so blinding is not applicable.

Reporting for specific materials, systems and methods

We require information from authors about some types of materials, experimental systems and methods used in many studies. Here, indicate whether each material, system or method listed is relevant to your study. If you are not sure if a list item applies to your research, read the appropriate section before selecting a response.

Materials & experimental systems

n/a	Involved in the study	
x		Antibodies
x		Eukaryotic cell lines
x		Palaeontology and archaeology
x		Animals and other organisms
	x	Human research participants
	x	Clinical data
x		Dual use research of concern

Methods

n/a	Involved in the study	
x		ChIP-seq
x		Flow cytometry
x		MRI-based neuroimaging

Human research participants

Policy information about studies involving human research participants

Population characteristics	In this study among overall participants, 17% were 18-35 years old, 49% were 35-65 years old, and 34% were >65 years old. Approximately 35% participants were male and 84% were non-Hispanic white. Table 1 of the manuscript provides detailed information on age, race (white, Black, Asian, other race), sex (male, female), population density (quartiles), current smoking, work as a frontline healthcare worker, interaction with suspected or documented COVID-19, and history of diabetes, heart disease, lung disease, and kidney disease (each yes/no)
Recruitment	Our study population includes all participants enrolled in the COVID Symptom Study smartphone application ("app") from March 29, 2020 to July 16, 2020 in the U.S. Participants were recruited through general and social media outreach, as well as direct invitations from the investigators of long-running prospective cohorts to study participants. Since our cohort is not a random sampling of the population, there remains a possibility for selection or collider bias, reverse causality, or generalizability. We acknowledge the potential of reverse causality, such as COVID-19 symptoms leading to behavior changes, including social distancing or face mask use. Moreover, we acknowledge the potential of collider bias since our study relies on voluntary participation which may lead to a greater likelihood of participants with COVID-19 symptoms or those more likely to observe social distancing or face mask use to provide data. To minimize these potential biases, we conducted prospective analyses after excluding participants who had any symptom related to COVID-19 or who had tested positive for COVID-19 prior to start of follow-up. We also acknowledge that data collection through smartphone adoption has comparatively lower penetrance among certain socioeconomic groups and that participants of an app study may have differential likelihood of reporting symptoms. However, our inverse-probability weighted (IPW) analyses to provide weights to participants such that it study sample has similar age, sex, and race distribution as the US population, did not substantially differ from non IPW analysis.
Ethics oversight	Partners Human Research Committee (Institutional Review Board Protocol 2020P000909)

Note that full information on the approval of the study protocol must also be provided in the manuscript.

Clinical data

Policy information about clinical studies

All manuscripts should comply with the ICMJEguidelines for publication of clinical research and a completedCONSORT checklist must be included with all submissions.

Clinical trial registration	The protocol is registered with clinicaltrials.gov (NCT04331509)
Study protocol	Partners Human Research Committee (Institutional Review Board Protocol 2020P000909). It can be made available on request.
Data collection	Our study population includes all participants enrolled in the COVID Symptom Study smartphone application ("app") from March 29, 2020 to July 16, 2020 in the U.S. Participants were recruited through general and social media outreach, as well as direct invitations from the investigators of long-running prospective cohorts to study participants.
Outcomes	We used a previously published symptom based classifier that predicts COVID-19 as our primary outcome measure. (1) Primary outcome: Because a report of a positive COVID-19 test depended on access to testing during the times of shortage of test kits, and incorporates a variable delay between symptoms and testing, we used a previously published symptom-based classifier that predicts COVID-19 (Predicted COVID-19) as our primary outcome measure. (2) Secondary outcome: We used testing positive for COVID-19 as our secondary outcome measure. Participants were asked if they had been tested for COVID-19, and if yes, the results (none, negative, waiting, or positive).
